# VISUCHIR 2024 Ecological Benchmarking of French Private Centers Using a Perioperative Digital Therapeutic: Descriptive Early-Adopter Comparison

**DOI:** 10.2196/95473

**Published:** 2026-07-15

**Authors:** Bogdan Adrian Buhas, Eric Potiron, Truong An Nguyen, Benjamin Pradère, Justine Figurelli, Christophe Almeras, Jean-Baptiste Beauval, Gilles Pasticier, Fabien Vidal, Guillaume Ploussard

**Affiliations:** 1 Urology Department, Groupe hospitalier Diaconesses Croix Saint-Simon, 125 Rue d'Avron, Paris, 75020, France, 33 686084728; 2 Urology Department, Clinique Urologique Nantes-Atlantis, Saint-Herblain, France; 3 Urology Department, Clinique La Croix du Sud, Quint-Fonsegrives, France; 4 Gynecology Department, Clinique La Croix du Sud, Quint-Fonsegrives, France; 5 Urology Department, Clinique Tivoli-Ducos, Bordeaux, France

**Keywords:** digital therapeutic, perioperative care, benchmarking, administrative claims data, ambulatory surgery, ecological study

## Abstract

Using publicly available 2024 VISUCHIR (Visualisation de la Chirurgie) benchmarking indicators, we performed a descriptive ecological comparison of national private-sector values and 4 early-adopter French private departments implementing the Betty Coaching perioperative digital pathway; early-adopter departments showed a directionally favorable, unadjusted profile for same-day discharge, mean length of stay, and VISUCHIR-reported readmission-evolution indicators, without causal inference.

## Introduction

Digitally enabled perioperative pathways combine prehabilitation, education, electronic patient-reported outcome measures (ePROMs)/electronic patient-reported experience measures (ePREMs), and postdischarge telemonitoring. Patient-level evaluations of the Betty Coaching perioperative digital therapeutic have reported feasibility, ePROM adherence, recovery outcomes, and transitions from on-site to app-based prehabilitation and rehabilitation pathways [1-3]. Associations between personalized eHealth and recovery after major abdominal surgery were described in a multicenter randomized trial [4], and other independent studies described remote monitoring in oncology [5], the relationship between eHealth and patient participation [6], and smartphone app–supported postoperative monitoring after oncologic surgery [7]. Whether comparable patterns appear in French national administrative benchmarks is unknown. VISUCHIR (Visualisation de la Chirurgie)—the Agence Technique de l’Information sur l’Hospitalisation (ATIH) surgical observatory derived from Programme de Médicalisation des Systèmes d’Information and Classification Commune des Actes Médicaux (CCAM) data—benchmarks ambulatory organization and readmission vigilance [8-10]. Our narrow aim is to describe whether 2024 VISUCHIR system-level indicators differ at private departments adopting Betty Coaching versus national private-sector benchmarks, without causal inference.

## Methods

### Study Design

We conducted a descriptive, ecological, cross-sectional analysis of the publicly available 2024 VISUCHIR comparative extract (consulted February 1 to March 30, 2026) [7-9]. No patient-level data were used.

### Data Source Constraints

VISUCHIR releases only the same-day discharge (SDD) percentage with its denominator, the mean inpatient length of stay (no dispersion), the 2024 caseload, and a derived 5-year relative change of the 30-day readmission rate; per-case data, medians, IQRs, CCAM-level breakdowns, and case-mix–adjusted indices are not released [8,9].

### Intervention

Betty Coaching delivers prehabilitation, rehabilitation, timed education, and checklists.

### Exposed Centers and Implementation

In total, 4 early-adopter private departments across 3 French private hospitals (Clinique Urologique Nantes-Atlantis; Clinique La Croix du Sud, urology and gynecology departments; and Clinique Tivoli) used Betty Coaching as the default perioperative pathway throughout 2024 for all eligible adult patients in their VISUCHIR denominators (intention to implement). Per-patient app uptake, adherence, and engagement cannot be extracted from VISUCHIR; patient-level adoption and adherence data for the same platform were reported in prior publications [1-3].

### Comparator

We used the 2024 VISUCHIR national private-sector benchmark, which, by construction, includes the four exposed centers; no exclusion-adjusted estimate was computed (this would require values not part of the VISUCHIR-published dataset).

### Outcomes and Procedure Groupings

The three prespecified VISUCHIR groupings, as defined by ATIH-published CCAM act bundles [8,10], were (1) all urological surgeries (the full CCAM list of urological acts: endoscopic, ablative, reconstructive, robotic, and oncologic procedures), (2) oncologic urology (principal oncologic CCAM acts: radical prostatectomy, radical and partial nephrectomy, and radical cystectomy), and (3) oncologic gynecology (hysterectomy and adnexectomy for malignancy, ovarectomy for malignancy, and pelvic or para-aortic lymphadenectomy). The exhaustive per-grouping act lists are published on the VISUCHIR platform [8,10]. The outcomes were SDD rate (discharge on the day of surgery), mean inpatient length of stay (days), 2024 caseload, and 5-year evolution of the 30-day cumulative readmission indicator.

### Statistical Analysis

VISUCHIR-released aggregate values were summarized descriptively, and no inferential statistical testing was performed. Absolute differences were calculated directly from the displayed VISUCHIR values to aid interpretation; they are reported in percentage points for SDD and readmission-evolution indicators and in days for mean length of stay. No *P* values, CIs, or model-based estimates were calculated because patient-level data, clustering structure, case-mix variables, and period-specific readmission numerators and denominators were unavailable.

### Ethical Considerations

This study was reviewed and approved by the local ethics committee of Clinique La Croix du Sud (IRB00010835) and was conducted in accordance with the Declaration of Helsinki. Only publicly available aggregated VISUCHIR (ATIH) indicators were used; no individual patient-level data were accessed, and no patient was identifiable. The requirement for written informed consent was waived by the ethics committee because the analysis involved only anonymized, publicly available, aggregated benchmarking data with no individual patient-level information.

## Results

Across the three groupings, the VISUCHIR-published SDD rates at the four early-adopter centers were higher than the national private-sector benchmarks, with descriptive absolute differences ranging from +5.9% to +11.6%. The mean inpatient length of stay was shorter at the early-adopter centers, and the VISUCHIR-reported 5-year evolution of the 30-day readmission indicator was directionally more favorable (Table 1).

**Table 1.** 2024 VISUCHIR (Visualisation de la Chirurgie) benchmarking indicators in French private-sector surgical activity versus 4 early-adopter centers using the Betty Coaching perioperative digital pathway. All France and Betty Coaching values are reproduced verbatim from the publicly available 2024 VISUCHIR comparative extract [8]. Absolute differences were calculated descriptively as Betty Coaching center values minus the France private-sector benchmarks. No inferential statistical testing was performed.

Surgical groups and indicators		France, private sector	Betty coaching centers	Absolute difference
All urological surgeries				
Same-day discharge rate, %		51.5	59	+7.5
Inpatient hospital stay^a^, mean number of days		3.6	2.6	−1
30-day readmission rate (VISUCHIR 5-year evolution)^b^, %		−4.3	−19.6	−15.3
2024 caseload, n		1,012,669	12,291	—^c^
Oncologic urology				
Same-day discharge rate, %		31.8	43.4	+11.6
Inpatient hospital stay^a^, mean number of days		4.4	3.2	−1.2
30-day readmission rate (VISUCHIR 5-year evolution)^b^, %		−0.6	−28	−27.4
2024 caseload, n		205,796	2697	—
Oncologic gynecology				
Same-day discharge rate, %		48.3	54.2	+5.9
Inpatient hospital stay^a^, mean number of days		7	4.4	−2.6
30-day readmission rate (VISUCHIR 5-year evolution)^b^, %		−6	−20.4	−14.4
2024 caseload, n		89,966	596	—

a VISUCHIR releases only the aggregated mean inpatient length of stay, without dispersion or individual-level data.

b VISUCHIR-defined relative change over 5 years for the 30-day readmission indicator; the underlying period-specific numerators and denominators are not released. The France benchmark includes the four exposed centers by construction.

c Not applicable.

## Discussion

In this descriptive, VISUCHIR-based ecological comparison, the four early-adopter departments using Betty Coaching had a favorable, unadjusted benchmarking profile in 2024, with higher SDD rates, shorter mean lengths of stay, and directionally more favorable readmission-related VISUCHIR indicators when compared to national private-sector benchmarks. These findings represent system-level administrative observations, not evidence that the digital therapeutic independently changed outcomes. Because this study is ecological, cross-sectional, nonrandomized, and unadjusted, its contribution is limited to identifying a hypothesis-generating, early-adopter signal across the three VISUCHIR surgical groupings.

These findings are consistent with, but do not validate, prior platform evaluations that reported feasibility, ePROM/ePREM adherence, and favorable recovery after urologic surgery [1-3]. They also align with broader independent evidence on personalized eHealth, smartphone-supported postoperative monitoring, and remote monitoring as components of perioperative care that may support recovery surveillance and patient-clinician coordination [4-7]. Our analysis extends this literature only at the administrative benchmarking level, by examining whether center-level implementation is accompanied by directionally coherent indicators.

Several limitations are central to interpretation. Early-adopter centers may already have been high-performing centers before implementation because of established ambulatory infrastructure, minimally invasive or robotic pathways, enhanced recovery after surgery protocols, discharge coordination, coding practices, or patient selection (“healthy adopter” bias). Moreover, the national private-sector benchmark includes the exposed centers; the comparison is therefore not a strict exposed-versus-unexposed contrast. Further, VISUCHIR provides aggregated public indicators rather than patient- or procedure-level data, precluding case-mix adjustment, clustering-aware modeling, center-level longitudinal analysis, and robust readmission inference; therefore, the absolute differences reported herein should be interpreted only as descriptive contrasts, not as adjusted effect estimates. The length of stay is available only as an aggregate mean, and the 5-year readmission-evolution indicator is a derived VISUCHIR vigilance metric, not a patient-level binomial outcome. Per-patient app uptake and adherence cannot be extracted from VISUCHIR, with individual exposure data for the same platform reported elsewhere [1-3].

These results support a cautious conclusion: early-adopter centers using Betty Coaching displayed a favorable, unadjusted VISUCHIR benchmarking profile in 2024, but causality and independent effectiveness remain unproven. Future work should use implementation timing; patient-level exposure and adherence; procedure-level case mixes; standardized readmission indices or observed-to-expected ratios; and quasi-experimental designs, such as interrupted time series, matched-center comparisons, mixed-effects modeling, or difference-in-differences methods.

## Abbreviations

ATIH: Agence Technique de l’Information sur l’Hospitalisation
CCAM: Classification Commune des Actes Médicaux
ePREM: electronic patient-reported experience measure
ePROM: electronic patient-reported outcome measure
SDD: same-day discharge
VISUCHIR: Visualisation de la Chirurgie
